# 971 Enhancing Burn Care: An Examination of Nursing Roles in Goals-of-Care Discussions and Treatment Plan Development

**DOI:** 10.1093/jbcr/iraf019.502

**Published:** 2025-04-01

**Authors:** Natalie Applebaum, Esther Teo, Eva Regel

**Affiliations:** University of Arkansas for Medical Sciences; University of Arkansas for Medical Sciences, Arkansas Children’s Hospital; University of Arkansas for Medical Sciences, Department of Medical Humanities and Bioethics

## Abstract

**Introduction:**

Nurses play a critical role in treating and caring for patients with burn injuries. They often serve as frontline providers who engage with patients and families throughout their treatment and recovery. Nursing involvement and input in goals-of-care discussions and development of treatment plans is essential in delivering holistic, patient-centered care. However, the extent of their participation in these discussions varies across burn centers and is often influenced by institutional policies, clinical hierarchies, and clinicians’ individual practices.

**Methods:**

This presentation explores the findings of a single center survey of nurses conducted at a burn center caring for both the pediatric and adult patient populations. The survey examined several key aspects including nursing participation in end-of-life discussions as well as effective communication of patient information and management suggestions to the physician team.

**Results:**

Our findings emphasize the importance of including nurses in developing treatment plans for burn patients, as their unique clinical insights and ongoing engagement with patients can enhance care coordination, improve patient outcomes, and foster more comprehensive, interdisciplinary care.

**Conclusions:**

The study results highlight the burn nurse’s role as a conduit of knowledge between the care team, patient, and family. Strategies to increase nurse participation in care team and patient discussions include institutional support for interdisciplinary care meetings, offering training to nurses on effective communication, providing access to clinical ethics and moral distress consultation teams, and ultimately promoting a culture that values the contributions of all team members.

**Applicability of Research to Practice:**

By encouraging nurse involvement in the treatment planning process, burn care units can improve patient-centered outcomes, promote cohesive care, and better address the multifaceted needs of both the pediatric and adult burn patient populations.

**Funding for the Study:**

N/A

